# The Comparison of Clinical and Histopathological Effects of Topical Psyllium (*Plantago ovata*) Powder and Silver Sulfadiazine on Second-Degree Burn Wound Healing in Rats

**DOI:** 10.29252/wjps.10.1.96

**Published:** 2021-01

**Authors:** Mohammad Jalilimanesh, Maryam Azhdari, Aghdas Mirjalili, Mohammad Ali Mozaffari, Seyedhossein Hekmatimoghaddam

**Affiliations:** 1 Herbal Medicine Research Center, School of Pharmacy, Shahid Sadoughi University of Medical Sciences, Yazd, Iran; 2 Department of Plastic and Reconstructive Surgery, Shohadaye Mehrab Hospital, Shahid Sadoughi University of Medical Sciences, Yazd, Iran;; 3 Nutrition and Metabolic Diseases Research Center, Ahvaz Jundishapur University of Medical Sciences, Ahvaz, Iran;; 4 Department of Nutrition, Faculty of Paramedicine, Ahvaz Jundishapur University of Medical Sciences, Ahvaz, Iran;; 5 Shahid Beheshti University Student Research Center, Tehran, Iran;; 6 Department of Advanced Medical Sciences and Technologies, School of Paramedicine, Shahid Sadoughi University of Medical Sciences, Yazd, Iran.

**Keywords:** Burn, Plantago ovata, Rat, Silver sulfadiazine, Wound

## Abstract

**BACKGROUND:**

Burn wounds are a worldwide health problem, leading to physical and psychological disabilities in all age’s groups. With regard to absorbent properties of *Plantago ovata* mucilage which can decrease wound moisture, we aimed to compare the effect of silver sulfadiazine (SSD) 1% and powdered *P. ovata* on second-degree burn wound healing in rats.

**METHODS:**

This experimental study was conducted on 30 male Wistar rats with second-degree burn in three groups. Group 1 (control) did not receive any treatment; group 2 and group 3 (treated groups) were dressed daily using SSD cream and *P. ovata* powder, respectively. The weight of rats, wound size (by applying ImageJ software) and percentage of wound healing on the 5^th^, 7^th^, 10^th^, 13^th^, 16^th^, 19^th^, and 22^nd^ days (by diagnosing a plastic surgeon) and histological cutaneous changes at day 22 were evaluated. The Prism software was applied for data analysis. The Haematoxylin & Eosin as well as Masson's trichrome staining were performed on wound skin biopsies.

**RESULTS:**

On day 22^nd^, 20%, 50% and 60% of the rats had complete wound healing in the control, SSD and *P. ovat*a groups, respectively. A significant decrease in wound size was shown in the treated groups compared to the control group (*P*<0.01), but no significant difference was shown between the treated groups (*P*>0.05).

**CONCLUSION:**

However, the wound healing in *P. ovata* group or SSD was better than the control group, and the significant difference was not found with the treated group.

## INTRODUCTION

Burn wounds are a worldwide health problem, which affects both developed and developing countries, leading to physical and psychological disabilities in all age groups ^1^. The use of medicinal herbs was used to heal the wounds such as diabetic foot, pressure sore, and also burn as a topical dressing around the world ^2^^, ^^3^, including Avicenna’s book named Canon of Medicine. Herbal medicine is generally cheaper than synthetic drugs ^4^.

Moreover, some current synthetic medicines used for topical therapy of burn wounds include silver sulfadiazine (SSD), mafenide acetate, silver nitrate, bacitracin, polymixin B sulfate, and hydrocolloid ^5^. Despite various literature about wound healing issues, this is still one of the medical challenges. Recently, it received more attention to find a drug or substance with fewer side effects and lower costs ^6^^, ^^7^. 

SSD 1% has many benefits such as wide-spectrum antimicrobial properties, low cost, and high tolerability, and is commonly used by most in burn wounds ^8^ and some burn hospitals (such as our center), though it can delay the process of wound healing because of its negative effect on the regeneration of keratinocytes ^9^. Other side effects of SSD include electrolyte imbalance, skin necrosis, skin discoloration, leukopenia, hyper-osmolality, methemoglobinemia, and hemolysis, liver and renal impairment ^10^, slower epithelialization, higher cost, more infections, and more pain ^1^.

*P. ovata* (psyllium) seed has been prescribed as a medicinal remedy for various discomforts since hundreds of years ago in the Iranian traditional medicine. Due to the pharmaceutical effects of *P. ovata*, foods fortified with its mucilage gain acceptance by their consumers. *P. ovata *or Plantago major L. is a common name for several members of this plant family, commercially utilized to produce mucilage ^11^. Its active components have already been identified, and* P. ovata* contains 19.8% mucilage ^12^.

The mucilage obtained from a group of herbal compounds yields a clear, colorless gel in contact with water. *P. ovata* mucilage is taken from its seeds ^13^^, ^^14^. *P. ovata* is used for musculoskeletal pain, gout, and skin sensitivity ^15^ in both traditional and modern treatments ^16^.

Due to the absorbent properties of *P. ovata* mucilage which can decrease wound moisture, and also other ingredients in this plant, we aimed to compare the effect of 1% SSD (as routine treatment) and powdered *P. ovata* on second-degree burn wound in rats. 

## MATERIALS AND METHODS

***Animals***

This animal trial was conducted on 30 male Wistar rats (mean weight: 300±20 g, mean age: 3-4 months). The rats were sourced from the animal house of the International Campus of Shahid Sadoughi University of Medical Sciences, Yazd, Iran, and the male sex was selected just for uniformity, without any postulated gender influence in terms of response to burn. Each rat was kept in a separate cage under standard environmental condition (12-h light/dark cycle, temperature ~ 23 ºC) and provided with standard laboratory food and ad libitum water. 

All the experimental procedures were confirmed by the research Ethics Committee of Shahid Sadoughi University of Medical Sciences, Yazd, Iran as well as international laws of animal rights, including the ARRIVE (Animal Research: Reporting In Vivo Experiments) guidelines and National Institutes of Health guide for the care and use of laboratory animals with following code:

(Ethical Code: IR.SSU.REC.1399.263)

***Burn Injury***

Each rat was initially weighed followed by general anesthesia via intraperitoneal injection of xylazine 5 mg/kg plus ketamine 50 mg/kg (Alfasan Co, The Netherlands), after which the area behind the animal's neck (which is inaccessible by the rat to scratch itself) was shaved completely ^17^. Then, the burn was made on the depilated area using a rectangular metal plate (2.1 × 2.55 cm) with an area of 5.35 cm^2^ at a temperature of 80 °C for one second. According to our experience and published literature, this procedure could result in a second-degree (superficial) burn ^18^. We did not measure the amount of pressure on the metal plate, but a plastic surgeon with experience in burn management confirmed that the burn is second-degree when the mentioned burn procedure was done. First-degree and third-degree burns were excluded from the study. 

***Treatment***

The rats were randomly divided into three groups of 10 and kept for 22 d (The day of burn creation was considered as the first day). Group 1 or the control group did not receive any treatments, and the burn was simply rinsed with physiological saline once every day. The burns in group 2 and group 3 were dressed daily using SSD 1% topical cream and *P. ovata* powder, respectively. The rats were kept under 12-hour darkness and 12-hour light without any food restriction. In groups 2 and 3, the burn wound was washed daily with physiological saline and dried by the sterilized non-woven gauze before the application of SSD or *P. ovata* powder. The amount of applied powder or SSD has been completely covering the burned area (for SSD a depth of at least 1 mm). 

***Plant Preparation***

*P. ovata* seeds were purchased from the local market of Yazd, with confirmation of herbarium voucher number by a botanist in the Yazd University, Iran. Then, they were milled (by stone mortar and pestle) to powder kept in a cool and dry place inside an enclosed glass jar throughout the course of the study (22 d). 

***Measurement of parameters***

***Weight***

The weight of rats was measured by an electronic scale with the nearest 0.01 g (WLC, Radwag, Poland).

***Wound Size***

The percentage of rats with either complete or partial objective wound healing was evaluated. Imaging of wounds was performed using a digital camera (Canon 1DS, Japan) with auto flash and 17-megapixel resolution on the days 1^st^, 5^th^, 7^th^, 10^th^, 13^th^, 16^th^, 19^th^_,_ and 22^nd^ days.

The surface area of wounds was measured by applying ImageJ software. Body surface area of rats was calculated by Meeh’s formula: A=9.83 W^2/3^, where: A=total body surface area (TBSA) in cm^2^, 9.83 is a constant, and W=weight in grams ^19^. The burned area's percentage of the TBSA (TBSA%) less than 15% in adults and less than 10% in children is called minor burn ^20^. TBSA% was calculated by the following formula: (Burned surface/A)×100. According to the above formula, the mean A and the TBSA% in rats were 447.54 cm^2^ and 1.2 %, respectively. 

During the 22-day course of treatment, the lack of healing, the percentage of partial healing, or complete healing in each group was evaluated by a burn reconstructive surgery specialist (plastic surgeon) who was the blindness of rats’ classification groups and it was recorded. 

***Tissue Staining***

On the final day (day 22^nd^), the rats were anesthetized to sacrifice with diethyl ether in a special container, and 6 rats were selected randomly and blindly (by a third person) from each group for histopathological analysis by scalpel blade. Burned skin with a little of the surrounding healthy skin was put in 10% formalin for fixation. After 48 h, the tissue processing (model DS 2028 / H, Did Sabz company, Iran) followed by microtome sectioning (model DS 8402, Did Sabz company, Iran) was performed to provide microscopic slides. The staining methods were haematoxylin-eosin (H & E) as well as Masson's trichrome ^21^.

***Histologic examination***

Microscopic examination of the histological changes in the skin (obtained on the 22^nd ^day) was performed by an experienced pathologist who was unaware of the treatment applied to each group of rats, using an optical microscope (model Magnum-T, Medline Scientific, UK). The four criteria of evaluation in H&E-stained sections included the integrity of the epidermis (repair by the proliferation of keratinocytes), dermal fibrosis, dermal angiogenesis (neovascularization), and the degree of neutrophilic infiltration in the dermis, with a comparison to the unaffected normal surrounding skin. Trichrome-stained slides were also assessed regarding the degree of fibrosis in the dermis. A subjective grading system of 4 bands (1+ = little, 2+ = mild, 3+ = moderate, 4+ = severe) was used to compare each histologic parameter, and the mean score of each parameter was obtained. For angiogenesis, if the number of new sprouting capillaries in each high power field of the microscope is very low, the angiogenesis is regarded little (1+); with increasing numbers of new capillaries (while comparing groups with each other), the grade would be higher, up to severe (4+). In this arbitrary grading method, 5 fields in burnt dermis areas were considered and their mean grade calculated. Due to the irregular shape of the capillaries, arterioles, and venules, it is not so justified to use software solutions such as ImageJ.

***Statistical Analysis***

The Prism statistical software was applied using one-way analysis of variance (one-way ANOVA), Tukey test (post-test), paired *t*-test. A *P*-value of less than 0.05 (*P*<0.05) was considered statistically significant.

## RESULTS

**Fig. 1:**
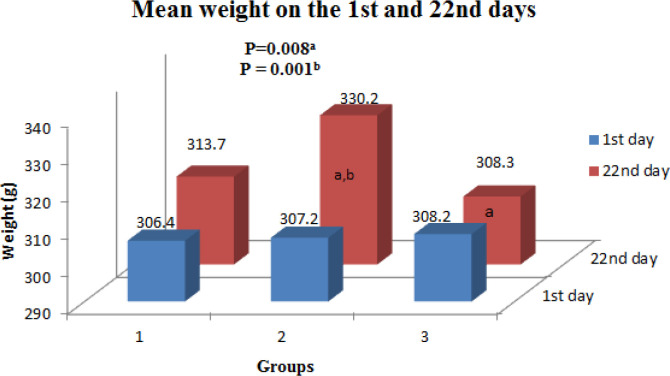
Mean weight on the 1^st^ and 22^nd^ days

^a^ The mean weigh of the group treated with the silver sulfadiazine cream was significantly higher on the 22^nd^ day compared to the *Plantago ovata* treated group.

^b^ The mean weight of the group treated with the silver sulfadiazine cream was significantly higher on the 22nd day compared to the first day

The rats’ mean weight was not significantly different in the three groups on the first day (*P*=0.95), whereas, on the 22^nd ^day, the mean weight was significantly different among the three groups (*P*=0.008). In addition, the mean weight in the group treated with SSD on the 22^nd^ day was significantly higher compared to the *P. ovata* treated group (Figure 1).

As is indicated in Table 1, the percentages of complete wound healing on the 22^nd^ day were 20%, 50%, and 60% in the control, SSD and *P. ovata* treated groups, respectively, which shows more healing in the SSD and *P. ovata* treated group in comparison with the control group. The difference between groups 2 and 3 was not significant (chi-square *t*-test: *P*>0.05). In the control group, one rat was not healed at all on the 22^nd ^day. The figures for the partial wound healing in each group on the 7^th^, 13^th^, and 22^nd ^days were shown in Figure A.1 (Appendix File). 

**Table 1:** The Percentage of rats with complete or partial wound healing on different days, in 3 study groups

**Group**	**7** ^th ^ **day**		**10** ^th^ ** day**		**13** ^th ^ **day**		**16** ^th ^ **day**		**19** ^th ^ **day**		**22** ^nd^ ** day**	
	**PH** ^b^	**CH** ^a^	**PH** ^b^	**CH** ^a^	**PH** ^b^	**CH** ^a^	**PH** ^b^	**CH** ^a^	**PH** ^b^	**CH** ^a^	**PH** ^b^	**CH** ^a^
Physiological saline n=10	0	0	0	30	0	90	0	90	10	80	20	70
Silver sulfadiazine n=10	0	30	0	60	0	80	20	80	30	70	50	50
*Plantago ovata* n=10	0	0	0	20	0	100	0	100	20	80	60	40

The partial or complete healing in each group was evaluated by a plastic surgeon (who was the blindness of rats’ the classification groups).

^a^ Complete wound healing (CH) means complete healing of the wound.

b Partial wound healing (PH) means the wound has been relatively recovered.

The mean of wound surface area (wound size) shows a gradual decrease in all groups, but was significantly different only on the 22^nd^ day (*P*<0.01). In both the SSD and *P. ovata* treated groups, better healing was seen in comparison with the control group. Despite the measurements on the 5^th^ day in this study, we did not show its data, due to its similarity with the first day (Table 2).

***Results of histological assessment***

 Microscopic examination of the histological changes in the skin was performed on H&E-stained sections and also in trichrome-stained slides regarding the degree of fibrosis, angiogenesis, and neutrophilic infiltration in the dermis on the 22^nd ^day. Moreover, the integrity of the epidermis (repair by the proliferation of keratinocytes) was checked.

**Table 2:** Wound size (cm^2^) on different days, in the studied groups

**Group**	**7** ^th ^ **day**		**10** ^th^ ** day**		**13** ^th ^ **day**		**16** ^th ^ **day**		**19** ^th ^ **day**		**22** ^nd ^ **day**	
	**SD**	**Mean**	**SD**	**Mean**	**SD**	**Mean**	**SD**	**Mean**	**SD**	**Mean**	**SD**	**Mean**	
Physiological saline (n=10)	5.35	0	5.11	0.39	3.03	1.43	1.73	1.05	1.08	0.71	0.70	0.51	
Silver sulfadiazine (n=10)	5.24	0.19	4.92	0.51	4.15	1.13	1.82	1.02	0.78	0.83	0.12	0.13	
Plantago ovata (n=10)	5.35	0	5.22	0.36	3.12	1.13	1.56	0.81	0.56	0.44	0.10	0.19	
*P* value	0.934		0.578		0.712		0.72		0.184		< 0.01^a^	

The wound size was measured by ImageJ software.

^a ^Mean of wound area was significantly lower in both the silver sulfadiazine and *Plantago ovata* treated groups in comparison with the control group.

**Fig. 2:**
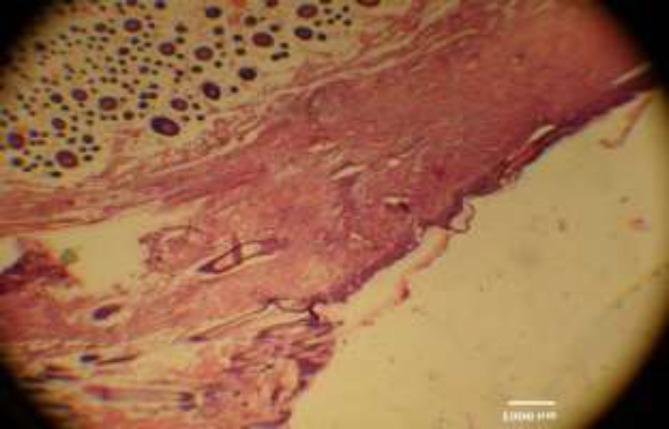
The burnt skin, with adjacent normal skin (40 ×, H&E staining)

**Fig. 3:**
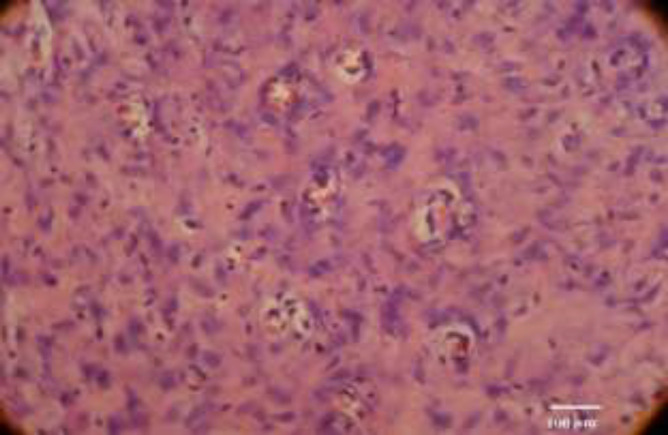
*Marked angiogenesis (400 ×, H&E staining)*

**Fig. 4:**
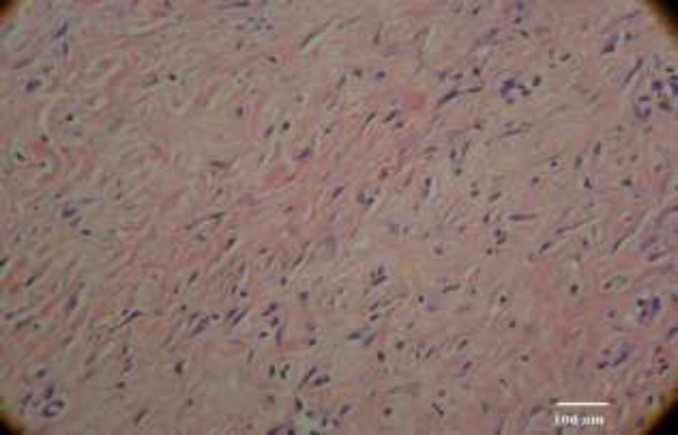
Complete healing by fibrosis (400 ×, H&E staining)

A small erosion (focal absence of the epidermis) was found in 2 (out of 30) rats (one in the control group and another one in *P. ovata* treated group), and larger ulceration of the epidermis in another 2 (similarly, one in the control group and another one in *P. ovata* treated group). These four samples, which had ulceration or erosion of epidermis, were excluded from the calculation of mean of histologic parameters since they were associated with marked neutrophilic infiltration and also due to the fact that they indicated traumatic injury after the intervention. Among the remaining rats, the findings are summarized in Table 3. Figures 2-4 are depicted as exemplary photomicrographs of the sections.

In terms of angiogenesis and neutrophilic infiltration, the results revealed no significant difference between the three groups (*P*=0.1, 0.25, respectively). However, the degree of fibrosis based on H&E as well as Masson's trichrome staining showed significantly higher values in the SSD and *P. ovata* groups in comparison with the control group (*P*<0.01), but without any difference between the SSD and *P. ovata* treated groups.

***Weighing of the healing indices***

To reach a general conclusion on the overall healing of burns, we used an arbitrary weighing system (1 to 10, based on presumed importance of each parameter) for all of the indices of wound repair, consisting of wound area reduction, subjective healing by observation, microscopic fibrosis (both on H&E-stained slides and on trichrome-stained slides), angiogenesis, and the neutrophilic infiltration, which was assessed on the final day of treatment, (the 22^nd ^day). For each parameter, the percentage of goal achievement (i.e., ideal complete healing) was multiplied by its weight. 

 The suggestive formulas for each index are as follows:

Wound area reduction (%)=1 - [mean of wound surface (cm^2^) on the 22^nd^ day / mean of wound surface (cm^2^) on the 1^st ^day] × 100

Subjective healing (%) = mean percentage of complete healing by observation

Fibrosis on H&E-stained slides= mean of fibrosis ×100/4

Fibrosis on Trichrome-stained slides= mean of fibrosis ×100/4

Angiogenesis= (4/mean of angiogenesis) ×100/4

Neutrophilic infiltration= (4/mean of neutrophilic infiltration) ×100/4

Table 4 shows the scores according to such arbitrary weighing of healing parameters. The sum indicates the overall performance of each type of treatment; the higher, the better.

**Table 3:** The mean and the percentage of Microscopic markers of healing ^a^

**Group **	**Angiogenesis**	**Neutrophilic infiltration**	**Fibrosis in H&E**	**Fibrosis in Trichrome**
Control; mean (%)^b^, n=4	3 (75)	1 (25)	2.25 (56.2)	2 (50)
Silver sulfadiazine; mean (%)^b^, n=6	2.5 (62.5)	1.33 (33.2)	3.66 (91.5)	4 (100)
*Plantago ovate*; mean (%)^b^, n=4	1.75 (43.7)	1.5 (37.5)	3.5 (87.5)	4 (100)
P-value	0.1	0.25	< 0.01	< 0.01

H&E,haematoxylin-eosin;

^a ^Microscopic markers of healing in 14 skin biopsies from the burnt rats.

^b^ The mean and the percentage of each parameter are evaluated based on grading 1+ to 4+.

**Table 4:** Scores of 3 groups according to the arbitrary weighing of healing parameters

**Group (each n=6)**	**Sum of wound area reduction**	**Sum of subjective healing**	**Angiogenesis**	**Neutrophilic infiltration**	**Fibrosis** **in H&E**	**Fibrosis** **in ** **Trichrome**	**Sum**
Ideal situation	100%	100%	0	0	4	4	-
Weight	7	9	4	2	8	10	-
Control	53.5	180	133.3	200	450	500	1516.8
Silver sulfadiazine	305.9	360	160	153.8	732	1000	2711.7
Psyllium	375.5	540	228.5	133.3	700	1000	2977.3

H&E, haematoxylin-eosin;

## DISCUSSION

Herbal remedies for the treatment of thermal injuries are a trend that is more or less popular in every country ^22^. Briefly, the findings of the present study were shown 1) a significant increase in the mean weight changes in the group treated with SSD (*P*=0.001). We have not been able to justify this weight gain difference (though all rats are growing naturally in this age, that is, 3-4 months); 2) according to the plastic surgeon’s diagnosis, 20%, 50% and 60% of the rats had complete wound healing in the control, SSD and *P. ovat*a groups at the end of the study, respectively; 3) using ImageJ software, wound healing were more in the SSD treated and *P. ovata* treated groups compared to control group, but no significant difference was shown between the SSD and *P. ovata* treated group; 4) based on microscopic markers of healing, a) angiogenesis was less in the *P. ovata* treated group compared to the control group; this means that treatments were effective in promoting tissue repair; b) according to the H&E as well as Masson's trichrome staining, the degree of collagens/ elastic/ reticulin tissue fibrosis, perhaps the most important indicator of long-term healing, was significantly more in the treatment groups (without any difference between the SSD and *P. ovata* treated groups) in comparison with the control group (*P*=0.00); c) the severity of the accumulation of neutrophils in the dermis was not so different among groups (*P*=0.25). 

 The overall performance of *P. ovata* was better than the SSD or physiological saline according to the novel method of calculation of various healing parameters which we introduce in this study.

The association of medicinal plants with wound healing has been proven. On the assessment of second-degree burn healing, the maximum healing belonged to the group treated with nettle extract, whereas the least healing was in the control group ^23^.

The superior healing effects of creams prepared from *quince seed* mucilage on dermal toxicity compared to no treatment or the *eucerin* cream without mucilage were shown. Mucilage can be used due to its high water absorption capacity which helps to reduce physical and chemical stimulation. Mucilage does not have any systemic absorption in comparison with other chemical drugs and medicines that may have systemic absorption and side effects. The proposed mechanisms of the action of mucilage are as follows: it may help in maintaining the proteins that cover the surface of the wound, as a physical barrier which reduces the evaporation of water, which may prevent the permeation of microorganisms, activating growth factors, facilitating the presence of fibroblasts and collagen production, simplifying the formation of the granulation tissue, directing more blood flow to the damaged tissue and improving wound healing ^24^.

The effect of three herbs was investigated (*Aloe vera, Robacin, *and* Rimojen*) on the simultaneous deep second degree and third-degree burns of 40 rats in comparison with SSD. Image-based results revealed a more efficient wound healing in the group treated with Robacin compared to other groups. In addition, the rate of wound healing in *A. vera* group was demonstrated to be higher than Rimojen and SSD groups. At the same time, the maximum healing of the second-degree burns was observed in *Rimojen* and *A. vera* groups. At histological analysis, the minimum speed of angiogenesis and fibrosis was attributed to the Robacin group, and less scar was observed in this group. Burn wound healing speed was also higher in the *Robacin* group ^4^. *A. vera* gel is extracted from the mucilaginous tissue. It is one of the constituents of some cosmetics and drugs used for burn wound healing in many countries. However, its clinical evidence remains unclear ^25^. In Kerman Burn Center, Iran, the effect of *A. vera* and 2% nitrofurazone ointment in the patients with second-degree burns was compared and concluded that *A. vera* gel was superior in speeding up the wound healing ^26^. 

The effects of honey and SSD were compared in 150 patients of all ages having similar types of superficial and partial-thickness burns at two sites on different parts of the body were included. Each patient had one burn site treated with honey and one treated with topical SSD, randomly. The rate of re-epithelialization and healing of burns was significantly higher in the patients treated with Honey compared to the SSD treatment. The site treated with honey healed completely in less than 21 d versus 24 d for the site treated with SSD ^27^. All in all, moisture-retentive dressings are one of the new trends in treating wounds ^28^.

## CONCLUSION

Treatment with *P. ovata* powder seems to result in remarkably more healing for second-degree burns compared to the control group in terms of wound healing and fibrous healing and also regarding the overall score of healing, although the wound healing effects of *P. ovata* were similar to SSD. The positive effects of *P. ovata* may be due to constituents of mucilage such as polysaccharides, tannin, coloring materials, pectin, plantagin, salicylic acid, carboxylic phenolic acids, flavonoids, minerals, etc. 

To conclude on the therapeutic effects of *P. ovata* powder for the treatment of second-degree burn wounds, more detailed comprehensive studies using individual components of *P. ovate* are needed. Moreover, a comparison of *P. ovata* powder with other commonly used medicinal plants harboring mucilage properties is recommended. Our proposed method of multiparametric assessment of burn wound healing is presented as a suggestion for future works on clinical/histopathologic aspects of burn repair. Besides, the findings could theoretically be similar in humans, studied. 
